# Cognitive Rehabilitation for Executive Dysfunction in Parkinson's Disease: Application and Current Directions

**DOI:** 10.1155/2012/512892

**Published:** 2011-11-02

**Authors:** Jessica Calleo, Cristina Burrows, Harvey Levin, Laura Marsh, Eugene Lai, Michele K. York

**Affiliations:** ^1^Mental Health Care Line, Michael E. DeBakey Veterans Affairs Medical Center, 2002 Holcombe Boulevard, Houston, TX 77030, USA; ^2^Department of Psychiatry, Baylor College of Medicine, One Baylor Plaza, Houston, TX 77030, USA; ^3^Parkinson's Disease Research Education and Clinical Center, Michael E. DeBakey Veterans Affairs Medical Center, 2002 Holcombe Boulevard, Houston, TX 77030, USA; ^4^Department of Physical Medicine and Rehabilitation, Baylor College of Medicine, One Baylor Plaza, Houston, TX 77030, USA; ^5^The Methodist Hospital Neurological Institute, Scurlock Tower 802, 6560 Fannin, Houston, TX 77030, USA; ^6^Department of Neurology, Baylor College of Medicine, 6550 Fannin, Suite 1801, Houston, TX 77030, USA

## Abstract

Cognitive dysfunction in Parkinson's disease contributes to disability, caregiver strain, and diminished quality of life. Cognitive rehabilitation, a behavioral approach to improve cognitive skills, has potential as a treatment option to improve and maintain cognitive skills and increase quality of life for those with Parkinson's disease-related cognitive dysfunction. Four cognitive rehabilitation programs in individuals with PD are identified from the literature. Characteristics of the programs and outcomes are reviewed and critiqued. Current studies on cognitive rehabilitation in PD demonstrate feasibility and acceptability of a cognitive rehabilitation program for patients with PD, but are limited by their small sample size and data regarding generalization of effects over the long term. Because PD involves progressive heterogeneous physical, neurological, and affective difficulties, future cognitive rehabilitation programs should aim for flexibility and individualization, according to each patient's strengths and deficits.

## 1. Introduction

Cognitive dysfunction is a nonmotor feature of Parkinson's disease (PD) that contributes significantly to disability, caregiver strain, and diminished quality of life over the course of the disease [1, 2]. While there is not a “signature” deficit that characterizes cognitive dysfunction in PD, common features include executive dysfunction, visuospatial dysfunction, and short-term memory deficits. Even in the earliest stages of PD, cognitive decline, and executive dysfunction in particular, is present in up to a third of patients [3]. An estimated 50% of individuals with PD have mild cognitive impairments in the absence of dementia [4, 5], and 25–30% of individuals with PD meet criteria for dementia [6, 7].

With advances in medical and surgical interventions for motor symptoms, individuals with PD are living longer and facing greater disability related to cognitive impairments. Accordingly, there is a corresponding need to address cognitive changes therapeutically. Yet, to date, there are no definitive treatments for cognitive dysfunction in PD [8]. Medication trials, aimed at slowing progression of cognitive decline or improving cognitive performance, have had variable success in decreasing functional impairment [8, 9]. Alternative or adjunctive behavioral interventions for cognitive dysfunction have the potential to reduce disability and improve quality of life in individuals with PD and their caregivers. Originally developed to improve cognitive functioning after traumatic brain injury (TBI), cognitive rehabilitation programs have recently been adapted for other neurological conditions [10]. However, there are no standardized guidelines regarding the types of strategies that offer the most beneficial outcomes, or the types of cognitive impairments or stages of cognitive decline for which treatment is most beneficial that would guide application in PD. 

Cognitive rehabilitation, a behavioral treatment approach for individuals with cognitive dysfunction, is designed to reduce functional impairment and increase engagement in daily adaptive activities. Although there is variation across programs, the essential elements of cognitive rehabilitation consist of basic skills training related to performance of vocational, social, and adaptive daily living skills. Subsets of cognitive training programs target improvements in specific cognitive domains, including visuospatial awareness, attention, working memory, or executive functioning, which are the essential cognitive skills to complete daily living tasks. Cognitive rehabilitation strategies consist of restorative or compensatory techniques. Restorative techniques focus on strategies to improve cognitive functioning to closer to the patient's level before there was an obvious decline. Specific restorative skills include techniques to improve recall of information over increasing periods of time (spaced retrieval) or using less intense cues (vanishing cues), computerized drills and repeated prompting to improve memory and attention and recall of remote memories (reminiscence therapy). Compensatory techniques provide strategies that organize information to improve recall and learning and provide instruction in self-management strategies. Compensatory techniques also include using multiple senses to improve learning and retrieval, procedural training to learn increasingly more complex behaviors, and external cues such as memory notebooks or calendars to improve recall. Programs may also teach, in-person or with the aid of computerized devices and software, strategies to improve self-management, such as problem solving, time management, and compensation for impaired memory [11]. Examining which cognitive strategies have the most beneficial impact on cognitive functioning, and adaptive living skills for PD could be the initial step in evaluating the feasibility and utility of cognitive rehabilitation programs in this population. 

The short- and long-term cognitive impairments following traumatic brain injury (TBI) are optimal targets for cognitive remediation, and positive outcomes following cognitive rehabilitation have been consistently demonstrated [10]. Standard TBI rehabilitation programs include training in strategies to compensate for attention deficits, visual scanning (for visual neglect), apraxia, language and functional communication, and mild memory deficits [12]. As TBI patients often have executive dysfunction, inclusion of emotional self-regulation and motivation skills into problem-solving skills has been shown to be effective in TBI cognitive training programs [10, 13]. While numerous cognitive rehabilitation programs have been developed for TBI, which is an acquired brain injury; less research has focused on progressive neurological disease.

 Cognitive rehabilitation programs for neurological disorders, including studies in Alzheimer's disease (AD), vary considerably in content (memory, learning, executive functioning, attention), administration (individual, group, computerized), timing (1 session versus 15 sessions) and setting (inpatient versus outpatient) [14, 15]. In Alzheimer's disease (AD), the studies have used primarily restorative strategies with both individual and group cognitive training programs have demonstrated positive outcomes [14, 15]. These cognitive training programs in AD provide evidence that patients with progressive neurological conditions can benefit from retraining. However, memory-training strategies are the primary cognitive skill evaluated in AD and rehabilitation studies have not evaluated executive functioning in this population. 

Lack of agreement regarding the definitions of PD-cognitive dysfunction and procedures (e.g., structured interviews, expert consensus, or neuropsychological tests) that best identify cognitive changes and impairments is a major challenge to treatment. This is particularly evident in PD-Mild Cognitive Impairment (PD-MCI), which has been defined inconsistently across studies. In general, MCI refers to an intermediate severity of acquired cognitive dysfunction between the cognitive status of “within normal limits” and “demented.” Whereas the MCI classification was developed and validated for conversion to Alzheimer's disease (AD) [16], similar nomenclature and criteria were proposed for PD-MCI [17].

Prevalence of PD-MCI and more subtle cognitive dysfunction in PD ranges from 21% to 55%, depending on the duration of the disease and which PD-MCI criteria and neuropsychological measures were used [4, 17, 18]. PD-MCI criteria have been outlined as the presence of a subjective report of cognitive problems by the patient or caregiver and performance at least 1.5 standard deviations below the age-corrected mean score in one cognitive domain without impairments in activities of daily living [17]. On the basis of these criteria, 21% of PD patients met criteria for PD-MCI and 17% met criteria for PDD [17].

In patients with PD-MCI, executive dysfunction/attention and memory impairment are the most prevalent deficits reported [4, 17]. By contrast, when PD-MCI is defined as performance that is only one standard deviation below the age-corrected mean across several neuropsychological measures [19], 53% of PD patients were classified as PD-MCI and the amnesic subtype was most prevalent, followed by the executive function and visuospatial subtypes. However, neither of these PD-MCI criteria identifies individuals whose test performance is significantly declined from a higher premorbid baseline but has yet to fall one or more standard deviations below normative test performance. 

 Cognitive deficits are noticed in daily life when they result in impaired performance in job-related duties, activities of daily living, and other activities that contribute to an individual's level of independence and well-being. For example, safety, financial planning and paying bills, driving, and occupational performance can be of concern in patients who demonstrate impairments in executive functions [20–23]. Accordingly, an approach to classification of cognitive dysfunction that overcomes the limitations of normative assessments focuses on functional change. With this approach, a functional change in performance that does not meet the PD-MCI criteria is described as subtle cognitive dysfunction [24]. Thus, individuals who have normal psychometric performance but who use compensation strategies or increased effort to avoid the functional impact of cognitive deficits would be captured with this classification. It would be helpful to identify what types of impairments from subtle to PD-dementia are most likely to benefit from cognitive rehabilitation. 

This integrative paper provides an overview of the types of cognitive impairments that are targeted in rehabilitation for cognitive dysfunction in PD and compares the content and delivery methods of cognitive rehabilitation interventions applied to patients with PD. Strengths and limitations of the current literature and future directions for cognitive rehabilitation in PD are discussed.

## 2. Methods

To identify relevant studies for the integrative review, keyword searches of abstract and titles were conducted in the PubMed and PsycINFO databases for studies published prior to July 2011. We used search terms (1) “Parkinson's disease” and “cognitive training”, (2) “Parkinson's disease” and “cognitive rehabilitation”, (3) “Parkinson's disease” and “cognitive remediation,” (4) “Parkinson's disease” and “training” and “executive.” Articles were included if they were original research in English, included individuals with Parkinson's disease, and described any type of intervention for cognitive functioning with pre- and postassessments.

## 3. Results

The PubMed search yielded a total of 18 unique articles and 3 additional studies were found in the PsycINFO database. Of the 21 abstracts reviewed, 17 were excluded. Reasons for exclusion were not reporting original research (e.g., review manuscripts, *n* = 5), not being in English (*n* = 2), not reporting cognitive rehabilitation interventions (e.g., interventions for gait, *n* = 6), not including pre- and postsassessments (*n* = 2), and subject population not individuals with Parkinson's disease (*n* = 2). 

### 3.1. Cognitive Rehabilitation in Parkinson's Disease

To date, there have been four reports of cognitive rehabilitation or training programs for patients with PD. Two are open-trial pilot studies, and two are small randomized controlled trials (RCTs) of cognitive rehabilitation programs targeting executive functioning, attention, and visuospatial abilities (see Table 1). 

In Sinforiani and colleagues' open trial [25], 20 patients with idiopathic PD who were enrolled in a day hospital motor rehabilitation program completed a computerized cognitive rehabilitation program that focused on improving attention, abstract reasoning, and visuospatial abilities. After cognitive training, PD patients had significantly improved verbal fluency, immediate and delayed logical memory, and visuospatial reasoning compared with their baseline assessments; these gains were maintained after 6 months. Although no differences were found after cognitive rehabilitation on measures of short-term memory, set-shifting or inhibition, this study suggests the potential for patients with PD to complete cognitive rehabilitation, improve performance on cognitive assessments, and maintain those gains. However, the small sample size, lack of a control comparison group, inpatient setting, and absence of a measurement of everyday functioning limit conclusions with respect to overall cognitive and functional improvement enhanced by the cognitive rehabilitation program and generalization of these findings to the larger population of PD patients who are not in an inpatient setting. 

Mohlman et al. [26] completed a small (*n* = 14) open trial to test the feasibility of an attention process training intervention for patients with a minimental state exam (MMSE) score >23 and idiopathic PD. The intervention consisted of in-person training with practice exercises and worksheets on attention tasks. Daily at-home practice exercises were also encouraged for all participants. Fourteen participants completed the program, and the self-report ratings on feasibility yielded positive results. The average rankings were between “some” to “much” perception of progress in improving their attention and enjoyment. The author reported that the participants improved on the measures of executive skills consisting of Digit Span backward, Stroop Color Word Test, Trail Making B, and Controlled Oral Word Association Test. This open trial successfully demonstrated the feasibility of administering an in-person cognitive training program and patients' acceptance of training. However, because of the small sample, study design and minimal outcomes measures, in addition to the lack of control group and long-term followup, conclusions as to the effectiveness of the intervention and translation into short-term or long-term functional outcomes are limited.

The two RCTs investigating cognitive rehabilitation in PD included relatively small sample sizes; however, these studies improved upon the literature by comparing the benefits of cognitive training programs to usual care received by individuals with PD. Sammer and colleagues [27] conducted an RCT for cognitive training of executive functions in the context of an inpatient rehabilitation program for PD. Participants were randomized to receive standard rehabilitation (occupational therapy, physiotherapy, and physical treatment) or standard rehabilitation plus a cognitive training program. The cognitive training program consisted of 10 sessions focused on facilitating working memory functioning, including search tasks, matrices, puzzles, speech production, picture completion, and storytelling. The 12 patients who completed the program demonstrated improved executive function compared with the 14 patients who completed only the standard treatment, even after controlling for premorbid intelligence, mood, age, dopaminergic medications, and disease severity. Additionally, the standard treatment arm had reduced working memory performance; whereas patients who received cognitive training maintained their baseline scores aftertreatment. This pilot RCT study was limited by a small sample size and lack of long-term assessments to evaluate maintenance of gains. Furthermore, the study did not address whether the treatment contributed to generalized improvements in daily activities or outside the inpatient setting. 

Another small RCT compared a 4-week outpatient computer-based cognitive training program to a speech therapy program matched on participation time [28]. All 33 participants had idiopathic PD and MMSE greater than 23. Among the subjects, 50% met criteria for MCI; but MCI was not a significant predictor in the outcome analysis. Following the 4-week intervention, the 18 participants in the cognitive training group demonstrated improved performance on attention, information processing, visual memory, verbal fluency, visuoconstruction, and executive functioning measures. Participants with and without MCI improved equally well-following treatment. The study had no long-term outcome assessments. 

## 4. Conclusions

Cognitive rehabilitation programs are increasingly recognized as beneficial alternatives to or adjunctive therapy for medications for improving specific types of cognitive dysfunction in patients with neurological disorders or maintaining patients at their current level; however, there is limited evidence for the effectiveness of cognitive rehabilitation in PD. The cognitive training programs for TBI and AD, which utilize the most well-developed programs have shown improvements in memory, attention, executive functioning, and problem solving [10], have demonstrated the feasibility of these retraining programs in either acquired or progressively deteriorating neurological conditions. Well-controlled, randomized larger scale investigations are needed for PD and other neurologically impaired population that take into account the specific disease characteristics of the population (e.g., duration of motor severity, medications), the specific cognitive domains affected in the population (e.g., executive dysfunction, visuospatial), the objective cognitive assessments, daily functioning assessments, and long-term outcome assessments. 

Although the current studies in PD are limited in sample size, it appears that cognitive training programs are both feasible and well accepted by PD patients. The cognitive targets of the reviewed pilot studies focus on attention and executive impairments in nondemented patients using both computers and in-person interventions. As the literature on cognitive rehabilitation programs grows, it would be beneficial for studies to use similar assessments to enhance comparisons between studies and to include both measures of neuropsychological and everyday functioning to evaluate the generalizability of the program into the patient's daily functioning. Based on the current literature, the effectiveness of cognitive training to demonstrate targeted short-term improvement on objective assessments following rehabilitation is promising [20–28]; however, long-term assessments of cognitive rehabilitation for patients with PD are needed. Both in-person and computerized training appear feasible but more information is needed on PD-related patient characteristics that predict success in cognitive rehabilitation interventions (e.g., age, length since diagnosis, type or severity of cognitive impairments). Executive dysfunction, which is an early indicator of PD-related cognitive decline and has a pervasive impact on daily functioning, has been identified as a target for cognitive rehabilitation in other populations. Accordingly, cognitive rehabilitation programs, particularly those that focus on improving executive functioning, have the potential to help patients with PD maintain a higher level of adaptive living skills and quality of life. 

Future cognitive rehabilitation outcomes studies in PD will need to address the limitations uncovered in programs developed for other neurologically impaired populations and the obstacles inherent in working with PD patients. Cognitive rehabilitation is often time-consuming for patients, caregivers, and therapists and can be costly to implement. In addition to these logistical obstacles is the lack of ecologically valid outcome measures to demonstrate improvement in patients' daily functioning or generalization of their newly acquired abilities to other areas of daily living. Well-controlled and described randomized studies using appropriate control groups with longer-term follow-up evaluations including ecologically valid outcome measures would be an initial step to demonstrate actual efficacy of cognition rehabilitation in PD. 

Additionally, PD itself poses several inherent obstacles for success in terms of cognitive rehabilitation. Researchers will also need to address these issues when developing future cognitive rehabilitation programs for patients with PD, including the heterogeneity of cognitive impairment, variability of functioning for patients with on/off fluctuations, cooccurring depression and anxiety, apathy, the mobility issues that restrict access to biweekly individualized programs, and the optimal disease stage in which improvements in cognitive functioning would be most beneficial. Personalized approaches to tailor treatment to individual strengths and deficits are recommended. If skills learned in cognitive rehabilitation carry over into everyday functioning and improve problem-solving and adaptive abilities, the programs could create positive and long-lasting benefits for patients by improving quality of life and potentially decreasing caregiver burden.

## Figures and Tables

**Table 1:** Cognitive training programs for patients with Parkinson's disease.

Author(s)	Total *N*	Randomized study	Length of treatment	Treatment	Cognitive targets	Outcome measures	Results
McKinlay et al. [24]	20	No	12 1-hour sessions over 6 weeks	Computerized software for neuropsychological training	Attention, abstract reasoning, visuospatial	Babcock's story, FAS, Raven matricies, Corsi-test, WCST and Stroop	PD patients improved on Babcock's story, FAS* and Raven matrices and at 6 months gains maintained. No differences from baseline on digit span, Corsi-test, WCST* and Stroop after training

Sinforiani et al. [25]	14	No	4 90-minute sessions over 4 weeks	Attention process training	Sustained, selective, alternating, and divided attention	Digits backward, Stroop, Trail Making Test B, FAS	Improvement on digits backward, Stroop, Trail Making Test B and FAS post treatment. On average, self-ratings were given for “some” to “much” progress, enjoyment and effort in the program

Mohlman et al. [26]	26	Yes 12 cognitive training 14 standard treatment	10 30-minute sessions during a 3-4 week rehabilitation hospital stay.	Working memory tasks	Executive functions	BADS	Cognitive training group significant improvement on BADS*

Sammer et al. [27]	33	Yes 18 cognitive training group15 control group	12 45-minute sessions over 4 weeks	Computerized software and paper-pencil exercises	Attention/ working memory, memory, psychomotor speed, executive functions and visuospatial	Digits Forward, Stroop, ROCFT, Semantic fluency, Trail Making B, TOL, PDQ-39 and CDS	Cognitive Training group had more improvement than Control Group after treatment on the Digit Span Forward, Stroop Word Test, ROCFT, Semantic fluency, Trail Making B and TOL. No group differences on the PDQ-39 or CDS

*Note: BADS: behavioral assessment of dysexecutive syndrome, FAS: phonological word fluency test; WCST: wisconsin card sorting task; ROCFT: Rey-Osterrieth complex figure test, TOL: tower of London, PDQ-39: Parkinson's disease questionnaire-39; CDS: cognitive difficulties in ADLs.
