# Erratum to “Cognitive Rehabilitation for Executive Dysfunction in Parkinson's Disease: Application and Current Directions”

**DOI:** 10.1155/2012/907513

**Published:** 2012-09-18

**Authors:** Jessica Calleo, Cristina Burrows, Harvey Levin, Laura Marsh, Eugene Lai, Michele K. York

**Affiliations:** ^1^Mental Health Care Line, Michael E. DeBakey Veterans Affairs Medical Center, 2002 Holcombe Boulevard, Houston, TX 77030, USA; ^2^Department of Psychiatry, Baylor College of Medicine, One Baylor Plaza, 6550 Fannin, Houston, TX 77030, USA; ^3^Parkinson's Disease Research Education and Clinical Center, Michael E. DeBakey Veterans Affairs Medical Center, 2002 Holcombe Boulevard, Houston, TX 77030, USA; ^4^Department of Physical Medicine and Rehabilitation, Baylor College of Medicine, One Baylor Plaza, 6550 Fannin, Houston, TX 77030, USA; ^5^Methodist Neurological Institute, Scurlock Tower No. 802, 6560 Fannin, Houston, TX 77030, USA; ^6^Department of Neurology, Baylor College of Medicine, 6550 Fannin, Suite 1801, Houston, TX 77030, USA

In this paper which appeared in *Parkinson's Disease* (2012, Article ID 512892), the reference citation numbers appearing by the authors' names in the left-hand side column did not match the correct references shown and cited. The reference numbers should appear as follows: for Sinforiani et al., the reference number has been changed from [24] to [25], Mohlman et al., the reference number has been changed from [25] to [26], Sammer et al., the reference number has been changed from [26] to [27], París et al., the reference number has been changed from [27] to [28].

## Figures and Tables

**Table 1:** Cognitive training programs for patients with Parkinson's disease (revised).

Author(s)	Total *N*	Randomized study	Length of treatment	Treatment	Cognitive targets	Outcome measures	Results
Sinforiani et al. [25]	20	No	12 1-hour sessions over 6 weeks	Computerized software for neuropsychological training	Attention, abstract reasoning, visuospatial	Babcock's story, FAS, Raven matricies, Corsi-test, WCST, and Stroop	PD patients improved on Babcock's story, FAS^∗^ and Raven matrices and at 6 months gains maintained. No differences from baseline on digit span, Corsi-test, WCST^∗^, and Stroop after training.
Mohlman et al. [26]	14	No	4 90-minute sessions over 4 weeks	Attention process training	Sustained, selective, alternating, and divided attention	Digits backward, Stroop, Trail Making Test B, FAS	Improvement on digits backward, Stroop, Trail Making Test B, and FAS posttreatment. On average, self-ratings were given for “some” to “much” progress, enjoyment, and effort in the program.
Sammer et al. [27]	26	Yes 12 cognitive training 14 standard treatment	10 30-minute sessions during a 3-4 week rehabilitation hospital stay.	Working memory tasks	Executive functions	BADS	Cognitive Training Group significant improvement on BADS^∗^
París et al. [28]	33	Yes 18 Cognitive Training Group 15 Control Group	12 45-minute sessions over 4 weeks	Computerized software and paper-pencil exercises	Attention/working memory, memory, psychomotor speed, executive functions and visuospatial	Digits forward, Stroop, ROCFT, semantic fluency, Trail Making B, TOL, PDQ-39 and CDS	Cognitive Training Group had more improvement than Control Group after treatment on the Digit Span Forward, Stroop Word Test, ROCFT, semantic fluency, Trail Making B, and TOL. No group differences on the PDQ-39 or CDS.

^∗^Note: BADS: behavioral assessment of dysexecutive syndrome, FAS: phonological word fluency test; WCST: Wisconsin card sorting task; ROCFT: Rey-osterrieth complex figure test, TOL: tower of London, PDQ-39: Parkinson's disease questionnaire-39; CDS: cognitive difficulties in ADLs.

